# Asthma control and its predictors in Ethiopia: Systematic review and meta-analysis

**DOI:** 10.1371/journal.pone.0262566

**Published:** 2022-01-13

**Authors:** Temesgen Mulugeta, Teshale Ayele, Getandale Zeleke, Gebremichael Tesfay

**Affiliations:** 1 Department of Clinical Pharmacy, School of Pharmacy, Institute of Health, Jimma University, Jimma, Oromia, Ethiopia; 2 Department of Pharmacology and Clinical Pharmacy, School of Pharmacy, College of Health Sciences, Addis Ababa University, Addis Ababa, Ethiopia; Srebrnjak Children’s Hospital, CROATIA

## Abstract

**Background:**

Determining the status of asthma control and identifying risk factors for poor asthma control is a key strategy for curbing the negative health impacts and the financial burden of the disease. Therefore, this review was aimed to determine the rate of asthma control and assess the predictors of uncontrolled asthma in Ethiopia.

**Methods:**

PubMed, Web of Science, and Google Scholar searches were performed using key terms; “asthma, bronchial asthma, control, controlled, uncontrolled and Ethiopia” up to October 16, 2020. University repositories were also searched to retrieve gray literature. The results were presented as a prevalence rate with a 95% confidence interval (CI). Subgroup analysis and meta-regression were performed to identify the sources of heterogeneity in the outcomes.

**Results:**

From 1,388 patients, based on the Global Initiative for Asthma (GINA) symptom control, the rate of the uncontrolled asthma was 45.0% (95% CI 34.0% - 56.0%) with a considerable heterogeneity between the studies; (I^2^: 94.55, p< 0.001). About 19.0% (95% CI 10.0% - 29.0%); (I^2^: 96.04, p< 0.001) of the asthma patients had a well-controlled asthma. Moreover, 36.0% (95% CI 22.0% - 50.0%), (I^2^: 97.11, p< 0.001) of patients had a partly controlled asthma. Similarly, based on the asthma control test (ACT), the rate of well-controlled asthma was 22.0% (95% CI 3% - 42.0%), with considerable heterogeneity between the studies; (I^2^: 97.75, p< 0.001). The most frequent predictors of uncontrolled asthma were incorrect inhalation techniques, frequent SABA use, moderate/severe persistent asthma, history of exacerbations, presence of comorbidities, use of oral corticosteroids, and irregular follow-up.

**Conclusion:**

The rate of uncontrolled asthma in Ethiopia was high. Several factors are associated with uncontrolled asthma. Comprehensive asthma educations at each follow-up visit should be strengthened to minimize the morbidity and the cost of uncontrolled asthma.

## Background

Asthma, the commonest chronic respiratory disease is usually characterized by chronic airway inflammation. It is a major cause of morbidity and mortality worldwide. In 2018, the World Health Organization (WHO) estimated that there were more than 339 million people with Asthma globally. Of these, 417,918 died within the same year, and most of the deaths were in Low and middle-income countries (LMICs) [1].

Asthma can cause multifaceted problems that negatively affect patients, their caregivers, and the country as a whole. It could result in work and school loss, poor quality of life, frequent emergency visits, hospitalizations, and death [2–4]. The economic impact associated with asthma is also stabbing. In 2016 alone, asthma was responsible for 24.8 million disability-adjusted life-years (DALYs) globally [1]. Recent data showed that medical expenditures attributable to asthma were significantly higher. Particularly individuals with uncontrolled asthma had up to 4.6-fold greater frequency of hospitalizations, 1.8-fold higher number of emergency department visits, and lower productivity, as they were more likely to be unemployed, more days absent from work and more activity limitations [5].

Asthma control and identification of risk factors for poor asthma control is a key strategy for curbing the aforementioned adverse health outcomes and the financial burden of the disease. The Global Initiative for Asthma (GINA) defines asthma control as the degree to which treatment has reduced the symptoms of asthma, and prevented disease exacerbations, worsening lung function, and drug side effects [6].

Studies conducted elsewhere on asthma control showed that majority of asthmatics have suboptimal control [7–10]. Notably, the asthma control and treatment (REACT) study conducted among US patients with moderate to severe asthma receiving standard asthma medications showed that asthma control was not achieved in 55% of the patients [11]. Similarly, a study conducted in Spain reported the prevalence of uncontrolled asthma as high as 63%. Treatment with oral corticosteroids (OCS), greater asthma severity, presence of distressing event, living in rural areas were associated with uncontrolled asthma [12]. A study by *Ghanname et al* noted that 29% of asthmatics had suboptimal control, and respiratory infections, concomitant diseases, animals allergy, adherence to treatment, health insurance and having more than two children were associated with asthma control [13].

With a high prevalence of asthma (8.7%) in Ethiopia (S1 Fig in S1 Annex), there are few discrete studies investigating the level of asthma control and its associated factors. In a study conducted at the capital of Ethiopia, 75.8% of asthmatic patients had uncontrolled asthma. The use of biomass fuel for cooking, longer duration of asthma, incorrect inhalation technique, and asthma exacerbation in the last 12 months was linked with uncontrolled asthma [14]. Similarly, *Zewdie at el* reported the prevalence of uncontrolled asthma to be as high as 50%. In this study, poor knowledge about asthma, a negative attitude about asthma, moderate asthma, and non-adherence to inhaled corticosteroids were associated with uncontrolled asthma [15].

So far, there is no aggregate data to determine the national burden of uncontrolled asthma and its predictors. Hence, we aimed this systematic review and meta-analysis to synthesize an evidence for the national prevalence of uncontrolled asthma and its predictors in Ethiopia. The finding of this study would have a valuable contribution for clinicians and policy makers to design appropriate strategy to tackle the problems associated with poor asthma control, which ultimately affects the quality of asthma care.

## Objective

This study aimed to estimate the rate of asthma control and the predictors of uncontrolled asthma among adult asthmatic patients in Ethiopia.

## Methods

This systematic review and meta-analysis was reported by Preferred Reporting Items for Systematic reviews and Meta-analysis (PRISMA) statement guideline for systematic reviews and meta-analysis and guided by PRISMA checklists [16]. This review did not have a protocol and hence not registered.

### Data sources and searching strategies

Two investigators (TM and TA) independently searched electronic databases; PubMed, Web of Science, and Google Scholar. Other sources such as Jimma and Addis Ababa University’s digital catalogs were also explored to retrieve unpublished documents. Electronic databases were searched using the combinations of the following key terms and Mesh terms along with the Boolean operators (“OR, AND”): “Asthma, bronchial asthma, control, controlled, uncontrolled, Ethiopia”. The searching was conducted from inception to October 16, 2020. The PubMed search detail was as follows: *((Asthma [Mesh] OR Asthma [tw] OR "bronchial asthma"[tw]) AND ("Prevention and control" [Subheading] OR control [tw] OR controlled [tw] OR uncontrolled [tw])) AND (Ethiopia [Mesh] OR Ethiopia [tw])*. Manual searches were also performed to find the reference lists of the selected articles. The PubMed search detail found at S3 Table in S1 Annex.

### Inclusion and exclusion criteria

All available articles reporting asthma control were included. Articles reporting patients with cardiac asthma were excluded. There were no articles that reported asthma control status in pediatric patients.

### Data extraction

A protocol for data extraction was designed by the authors and data were extracted independently by two authors (TM and TA). Any disagreements were resolved by consensus through discussion and by the authors (GZ and GT). Data were extracted on the name of the first author and year of publication, place of study, study design, participants’ sociodemographic, sample size, medication prescribed, asthma control status, and predictors of uncontrolled asthma.

### Outcomes definitions

The primary outcome of this systematic review and meta-analysis is rate of asthma control. It was reported either as per the Global Initiative for Asthma (GINA) control definition or Asthma control test (ACT) questionnaire. The secondary outcome was to determine the predictors for uncontrolled asthma.

As per the GINA guideline and other literatures [6, 14], asthma symptom control was classified as well controlled, partly controlled, and uncontrolled/poorly controlled. Well-controlled asthma was defined by the absence of daytime symptoms (no more than twice a week), the absence of nighttime symptoms, no limitations in activities, and limited need for rescue medication (not more than twice a week), and no exacerbations. Partially controlled asthma was present when daytime symptoms or rescue medication use was present more than twice per week and night waking or activity limitation was present in any week and exacerbations are present one or more per year. Uncontrolled asthma was defined as the presence of any three or more of these individual features within any week. According to the standardized self-administered ACT [6, 17], asthma control was classified as not well-controlled and well-controlled. Patients were classified as not well-controlled when the overall ACT score is less than or 19 and well-controlled when the overall ACT score is above 20.

### Assessment of quality of the studies

Two authors (GZN and GTD) independently appraised the quality of the included studies using the AXIS tool for Cross-sectional Studies [18] and Modified Newcastle Ottawa scale [19] for a prospective observational and case-control study. In the AXIS tool, for every correct answer, “yes” was assigned to each of the twenty questions. Otherwise, it was assigned “no” and not applicable (NA). The details of the quality appraisal results were found in S1 Table in S1 Annex.

### Data analysis and synthesis

We conducted a meta-analysis using OpenMetaAnalyst. Forest plots were used to estimate pooled prevalence with a 95% confidence interval (CI) to provide a visual summary of the data. To evaluate heterogeneity among studies, the Cochran’s Q test and I square (I^2^) indices were used. A significance threshold of p < 0.05 was applied to the heterogeneity (I^2^). At present heterogeneity, a random-effects model was used to compute the overall effect. A subgroup analysis was conducted to detect the impact of geographical regions on the status of asthma control. Meta-regression was also performed to assess the sources of the heterogeneity between the studies. We indexed a separate national prevalence of asthma and COPD to support the evidence (S1 and S2 Figs in S1 Annex).

## Results

### Study selection

**Fig 1:**
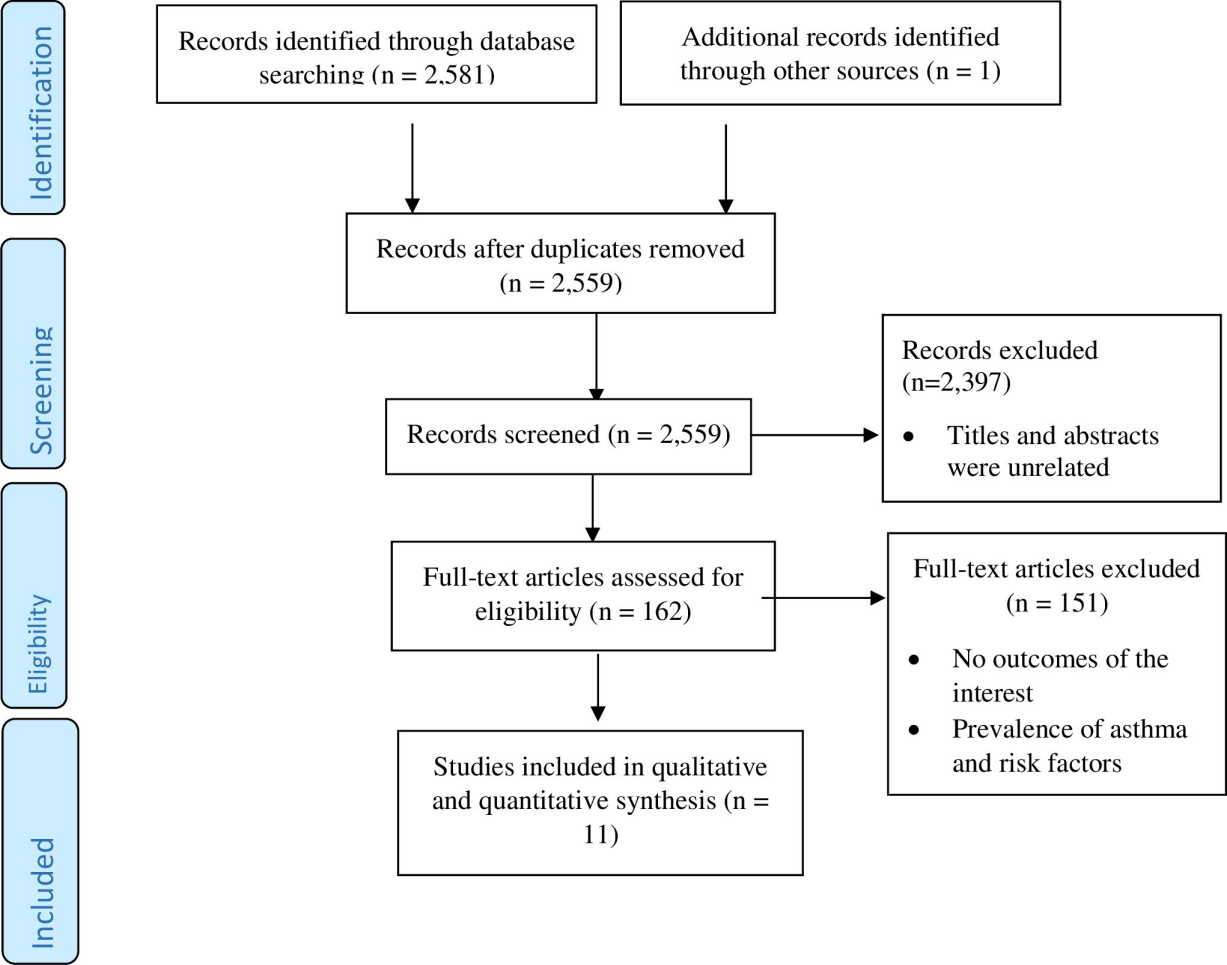
PRISMA flow diagram of the included studies.

The details of our search strategy were depicted in Fig 1. From all database searches, 2,582 records were identified. Twenty-three duplicates were removed keeping 2,559 records. Moreover, 2,397 records were removed because the titles and abstracts are unrelated to the outcomes of the review. The remaining 162 full articles were critically assessed for eligibility. Then, 151 articles were removed due to no reported outcomes of interest or asthma control. Finally, 11 full articles were selected and included in qualitative and quantitative analysis (Fig 1).

### Characteristics of the included studies

A total of 11 studies (2,501 patients, 57.06% females) were included in this systematic review and meta-analysis. Four studies each were from Jimma [15, 20, 22, 27] and Addis Ababa [14, 23, 25, 26], two from Gondar [17, 21], and one from Hosanna [24]. Nine studies [14, 17, 20–26] were cross-sectional record review along with patients’ interview, one was a prospective observational study [22] and the remaining one was case-control study [15].

All the included patients were attendants of outpatient department on follow-up for asthma. In all the studies, the minimum age was 18 years old except in a study by *Zemedkun et al* [20], where the minimum age of the participants was greater than 14 years old. In ten studies [14, 17, 20–27], the mean age of the patients was ranged from 35.1±9.4 years [24] to 54.46±10.01 years [26]. In five of the included studies [14, 17, 21–23], the mean durations of asthma since diagnosis ranges from 11.22±9.9 years [17] to 21.9 ±12.38 years [22]. From nine studies [14, 15, 20–24, 26, 27], 10.74% of the patients were smokers/ex-smokers. Among ten studies [14, 15, 17, 20–23, 25–27] with 2,327 patients, 27.37% of the patients had comorbid diseases. In five studies [15, 22, 23, 25, 27] with 993 patients, 32.32%, 36.86%, and 23.26% of patients had mild, moderate, and severe persistent asthma, respectively. In studies by *Tsegaye et al* [23] and *Zeru et al* [25], 21.74% and 13.60% of the patients had intermittent asthma, respectively. Two studies [14, 20] reported the Spirometry results. In studies by *Zemedkun et al* [20] and *Gebremariam et al* [14], among asthma patients who performed spirometry tests, 85.62% and 75.0% of the patients had FeV1 less than or equal to 80% predicted, respectively. In all the included studies, at least one controller medication was prescribed (Table 1).

**Table 1:** Characteristics of the included studies.

Authors, year	Study area	Study design	Sample size	Sex (n)		Age (mean), years	Duration of illness, (mean), years	Smoking (n)	Comorbidity (n)	Asthma severity (n)			
				Female	Male					Intermittent	Persistent		
											Mild	Moderate	Severe
Gebremariam et al, 2017 [14]	Addis	Cross -sectional	182	124	58	52 ± 12	20 ± 12.7	13	28	-	-	-	-
Zemedkun et al, 2014 [20]	Jimma	Cross-sectional	234	131	103	41.41±15.19	-	7	75	-	-	-	-
Mebrahtom et al, 2019 [21]	Gondar	Cross-sectional	206	119	87	47.41±13.207	15±13	13	28	-	-	-	-
Abegaz et al, 2020	Gondar	Cross-sectional	307	170	137	51.77 ±15.40	11.22 ± 9.92	-	164	-	-	-	-
Kebede et al, 2019 [17]	Jimma	Observational study	140	78	62	47.8 (19–74)	-	19	30	-	34	57	49
Zewdie et al, 2019 [15]	Jimma	Case-control	242	124	118	-	-	68	47	-	75	97	67
Fanta et al, 2016 [22]	Jimma	Cross-sectional	197	113	84	41.75 ±15.54	21.9±12.38	44	41	-	97	66	37
Tsegaye et al, 2019 [23]	Addis Ababa	Cross-sectional	230	150	80	54.3±15.1	12± 9.2	33	81	50	49	92	39
Dalo et al, 2017 [24]	Hosanna, SNNPE	Cross-sectional	174	65	109	35.1± 9.4	-	14	-	-	-	-	-
Zeru et al, 2020 [25]	Addis Ababa	Cross-sectional	184	80	104	44.1±13.6	-	-	65	25	66	54	39
Weldesenbet et al, 2018 [26]	Addis Ababa	Cross-sectional	405	273	132	54.46±10.01	-	5	78	-	-	-	-

### Asthma control based on the GINA symptom control

**Fig 2:**
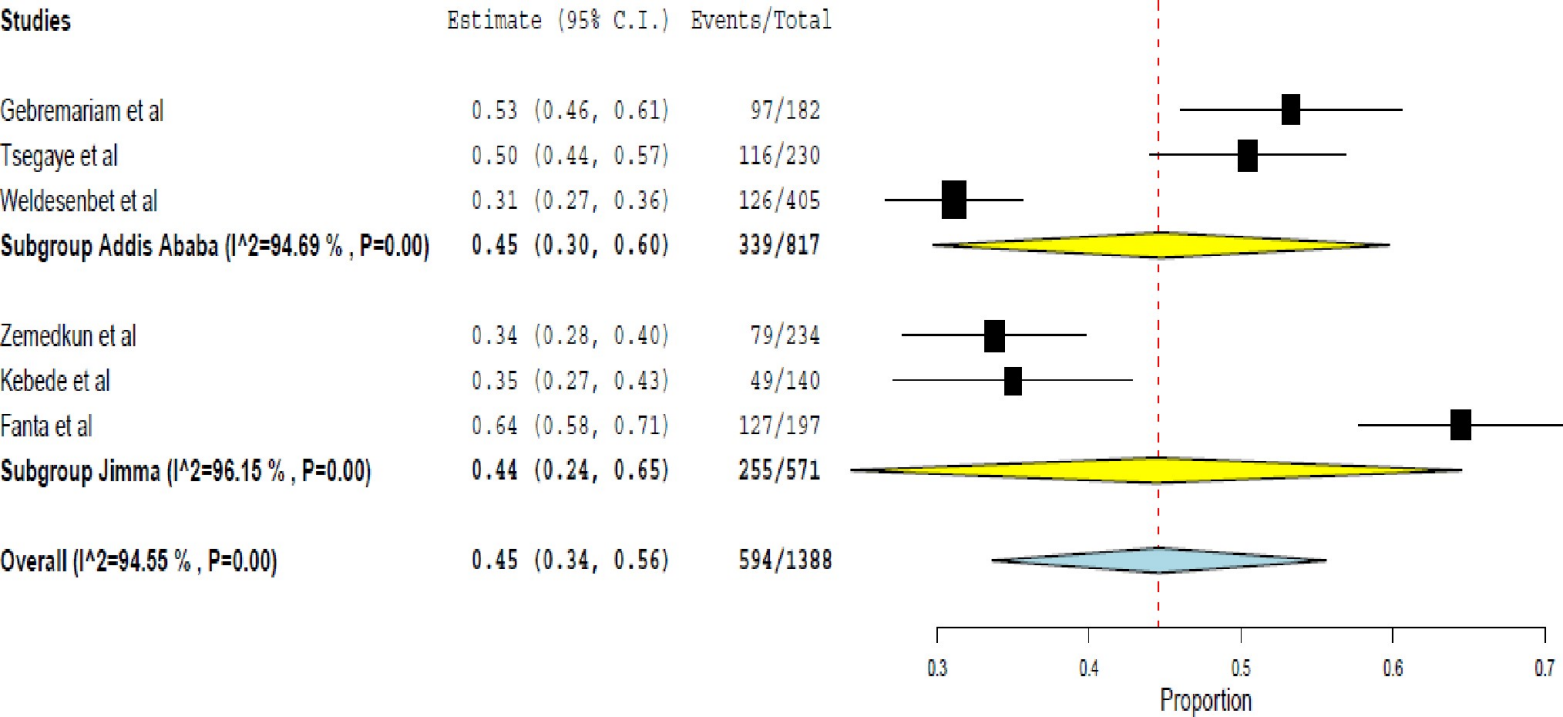
Rate of uncontrolled asthma assessed using the GINA symptom control.

A total of 6 studies [14, 20, 22, 23, 26, 27] were pooled, with 1,388 physician-diagnosed asthma patients. The result indicated, 45.0% (95% CI 34.0% - 56.0%) of the patients had uncontrolled asthma with a considerable heterogeneity between the studies; (Tau^2^: 0.02, Q (df = 5): 91.76, I^2^: 94.55, p< 0.001). Given the impact of geographical location on asthma control, a subgroup analysis was done based on the areas where the studies were actually conducted. Consequently, the rate of uncontrolled asthma in Jimma and Addis Ababa was 44.0% (95% CI 24.0% - 65.0%; Q (df = 2): 51.91 P< 0.001, I^2^ = 96.15%) and 45.0% (95% CI 30.0% - 60.0%); Q (df = 2) 37.70, P< 0.001, I^2^ = 94.69%), respectively (Fig 2).

**Fig 3:**
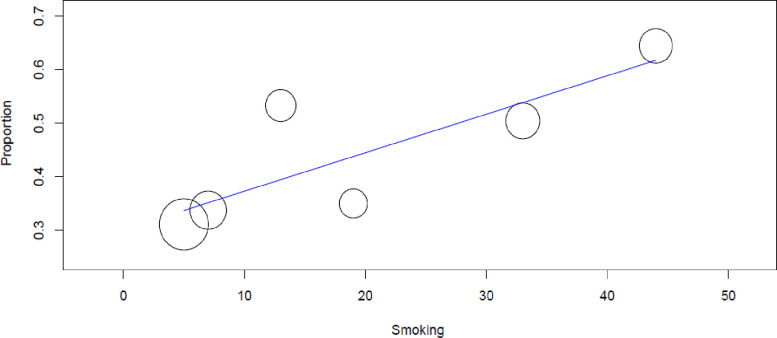
Regression plot of rate of uncontrolled asthma against smoking.

Meta-regression was performed with the covariates; the number of females, smoking, and comorbidity. The result showed smoking was associated with uncontrolled asthma (regression coefficients = 0.007 (95% CI 0.003–0.011), p< 0.001) (Fig 3).

**Fig 4:**
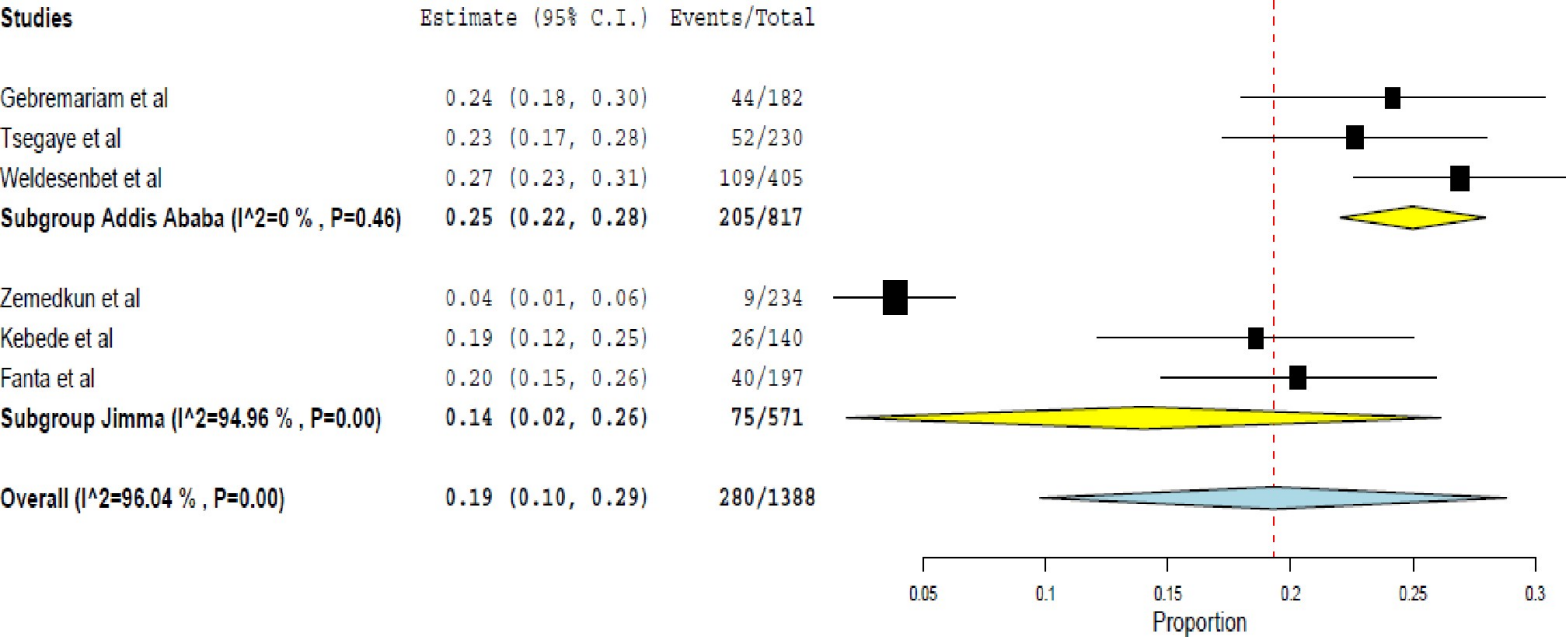
Rate of well-controlled asthma assessed using the GINA symptom control.

In this meta-analysis, the rate of well-controlled asthma was 19.0% (95% CI 10.0%-29.0%); I^2^ = 96.04%, p<0.001. The subgroup analysis showed the rate of well-controlled asthma was lower in Jimma; 14.0% (95% CI 2.0% - 26.0%) compared to in Addis Ababa; 25.0% (95% CI 22.0% - 28.0%)) (Fig 4).

**Fig 5:**
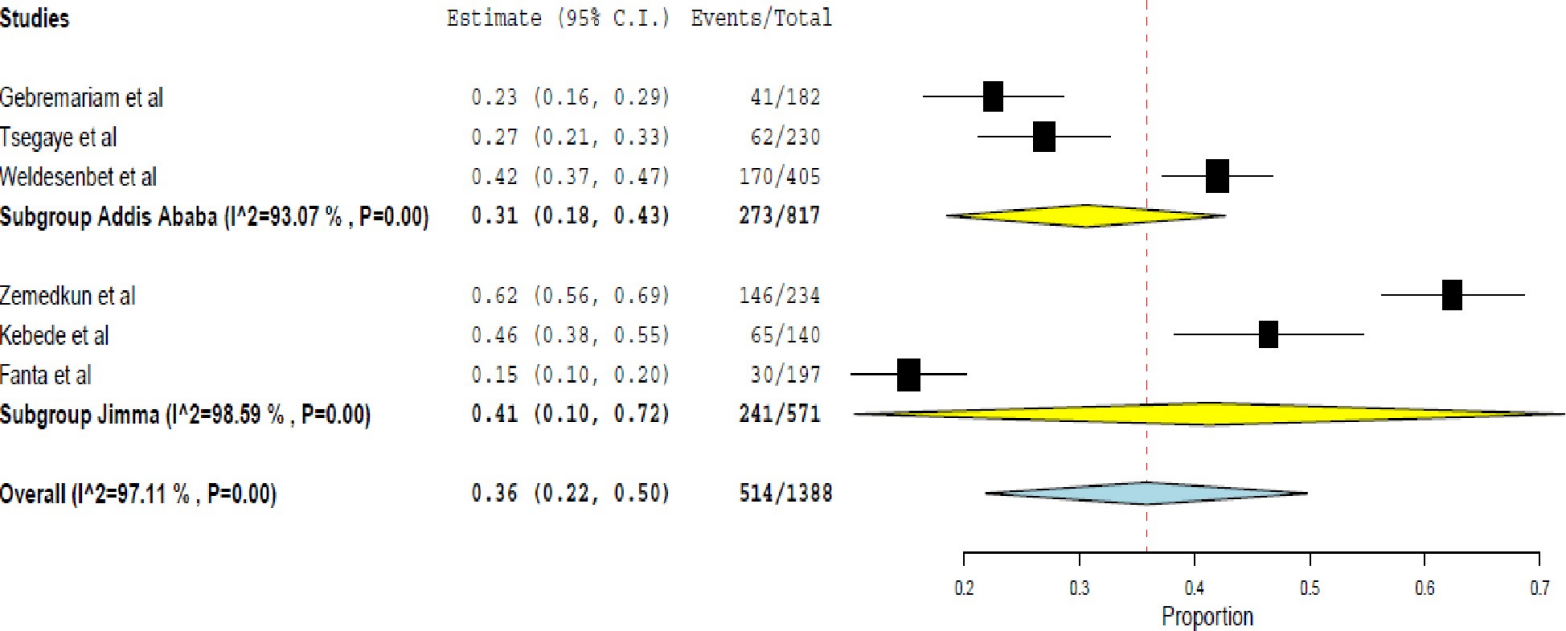
Rate of partly controlled asthma assessed using the GINA symptom control.

Moreover, the data generated from six studies [14, 20, 22, 23, 26, 27] showed, 36% (95% CI 22.0% - 50.0%) of asthmatic patients had partly controlled asthma. Heterogeneity among the studies (Q (df = 5) = 172.96, I^2^ = 97.11%, P< 0.001) (Fig 5).

Two studies [15, 24] were reported the asthma control as well-controlled and poorly controlled based on the GINA symptom control. In a study by Zewudie et al [15], 50% of the asthma patients were assessed to have poorly controlled asthma, while, in a study by Dalo et al [24], 56.2% of the asthma patients were assessed to have a poorly controlled asthma.

### Asthma control based on the Asthma Control Test (ACT)

**Fig 6:**
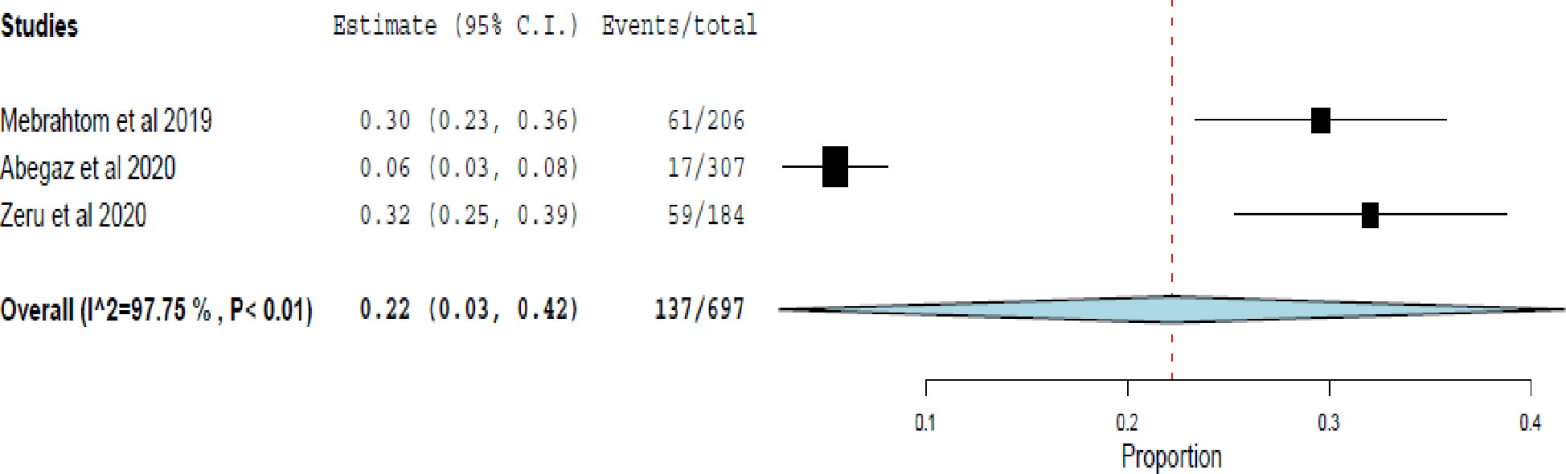
The rate of well-controlled asthma assessed using the Asthma Control Test (ACT).

The ACT tool was used in three studies [17, 21, 25] for assessing asthma control. ACT score ≥ 20 out of 25 total scores was used as a cut-point for well-controlled asthma. Among 697 asthma patients assessed, 22% (95% CI 3% - 42%) of the patients had well-controlled asthma. Considerable heterogeneity was detected among the studies (I^2^ = 97.75%, p<0.001) (Fig 6).

**Fig 7:**
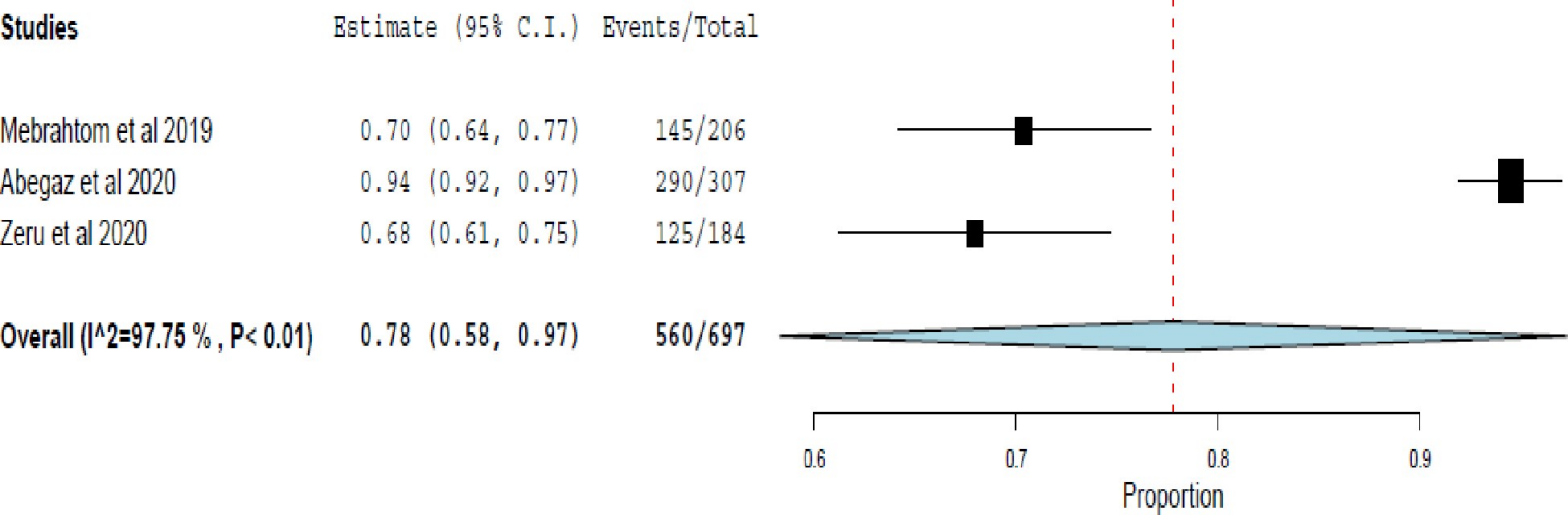
The rate of uncontrolled asthma assessed using the Asthma Control Test (ACT).

The remaining 78% (95% CI 58–97%) of the patients had uncontrolled asthma (Fig 7).

### Predictors of the uncontrolled asthma

In this systematic review, the predictors of the uncontrolled asthma adjusted for other variables were: incorrect inhalation techniques [14, 17, 21, 27], frequent SABA use [20, 22, 23], moderate/severe persistent asthma [15, 22, 23], history of exacerbations [14, 23, 27], comorbidities [22, 25, 26], use of oral corticosteroids (OCS) [20, 23], absence of regular follow-up [17, 20], use of biofuel for cooking [14], longer duration of asthma [14], perceived control of asthma [20], poor knowledge and negative attitude about asthma, non-adherence to inhaled corticosteroids (ICS) [15], low monthly income [22], being in the age category of 35–64 years [25] and cold weather [23] (Table 2).

**Table 2:** Diagnostic criteria and the outcomes of the studies.

Author, Years	Sample size	Asthma control	Diagnostic criteria	Associated factors of uncontrolled asthma
Gebremariam et al, 2017 [14]	182	Well controlled: 44	GINA symptom control	Longer duration of asthma (> 30 year), incorrect inhalation technique, asthma exacerbation in the last 12 months, and use of biomass fuel for cooking
		Partly controlled: 41		
		Uncontrolled: 97		
Zemedkun et al, 2014 [20]	234	Well controlled: 9	GINA symptom control	Unscheduled visit, Frequent inhaler SABA use, OCS use, Perceived control of asthma
		Partly controlled: 146		
		Uncontrolled: 79		
Mebrahtom et al, 2019 [21]	206	Well controlled: 61	ACT	Poor inhaler technique use
		Uncontrolled: 145		
Abegaz et al, 2020 [17]	307	Well controlled: 17	ACT	Not on a regular follow up and those not well competent on MDI use
		Uncontrolled: 290		
Kebede et al, 2019 [27]	140	Well controlled: 26	GINA symptom control	Use of asthma devices improperly and the presence of asthma exacerbation in the past 12 months.
		Partly controlled: 65		
		Uncontrolled: 49		
Zewudie et al, 2019 [15]	242	Well controlled: 121	GINA symptom control	Poor knowledge towards asthma, negative attitude towards asthma, moderate and severe asthma and non-adherence to ICS.
		Poorly controlled: 121		
Fanta et al, 2016 [22]	197	Well controlled: 40	GINA symptom control	Low monthly income, presence of comorbidity, moderate persistent asthma, severe asthma and use of SABA alone.
		Partly controlled: 30		
		Uncontrolled: 127		
Tsegaye et al, 2019 [23]	230	Well controlled: 52	GINA symptom control	Cold weather, history of exacerbations in last 12 months, moderate persistent, severe persistent, the use of SABA puff with Beclomethasone, and SABA puff with Beclomethasone and Prednisolone
		Partly controlled: 62		
		Uncontrolled: 116		
Dalo et al, 2017 [24]	174	Well controlled: 76	GINA symptom control	There is a strong association between asthma exacerbation and occupational status and use of social drugs.
		Poorly controlled: 98		
Zeru et al, 2020 [25]	184	Well controlled: 59	ACT	Age between 35–64 years and non-respiratory comorbidities.
		Uncontrolled: 125		
Weldesenbet et al, 2018 [26]	405	Well-controlled: 109	GINA symptom control	Asthma patients with depression were more likely to have uncontrolled asthma
		Partly controlled: 170		
		Uncontrolled: 126		

**Keys**: GINA: Global Initiative for Asthma, ACT: Asthma control test, SABA: Short acting bronchodilator, ICS: inhaled corticosteroid, OCS: oral corticosteroids, AOR: adjusted odds ratio, CI: confidence interval, MDI: metered dose inhaler, SNNPE: south nation, nationality and peoples of Ethiopia, n = number.

### Risk of bias

Studies were critically appraised using the AXIS tool (S1 Table in S1 Annex). Almost similar issues were identified in the domains of the appraisal tool. In many studies, the authors did not address the issue of non-responders, provide information, or categorize. In some of the studies, the selection of the participants was not representative of the source populations because convenience sampling was used, and it was not addressed how representative these samples were to the true population. In one study [22], the sample size for the participants was not justified. In a study by Dalo et al [24], since the design was retrospective, the issues of the non-respondents were described as “not applicable”. Two studies [15, 27] were critically appraised using the Modified Newcastle-Ottawa Quality Assessment Scale (S2 Table in S1 Annex). The quality of the two studies was moderate [15] and high quality [27].

## Discussion

The global epidemiological transitions are responsible for the changing burden of asthma in low and middle-income countries [28, 29]. In Ethiopia, the current national prevalence of asthma is 8.7% (95% CI 7.4–10.1%) (S1 Fig in S1 Annex). Although this figure is lower than the current national prevalence of COPD (18.3%) (S2 Fig in S1 Annex), it is causing a blistering attack on the health care system due to its substantial morbidity, mortality and higher health care costs [1, 30–32]. The key strategy for curbing such public health sufferings relies on proper asthma control and the identification of population-specific risk factors for uncontrolled asthma. Hence, this meta-analysis and systematic review summarized the rate of asthma control and predictors of uncontrolled asthma in Ethiopia.

This systematic review found 45.0% of uncontrolled asthma and 36.0% of partly controlled asthma in Ethiopia. Incorrect inhalation techniques, frequent SABA use, moderate/severe persistent asthma, history of exacerbations, presence of comorbidities, use of oral corticosteroids, and irregular follow-up were associated with uncontrolled asthma. In major cities; Jimma and Addis Ababa, the rate of uncontrolled asthma was 44.0% and 45.0%, respectively. Moreover, the study found much worrisome figure for the rate of well-controlled asthma. It was 19.0% at the national level, 14.0%, and 25.0%, respectively, in Jimma and Addis Ababa.

Despite the advances achieved in the development of new diagnostic tests like the fractional exhaled nitric oxide (FeNO), new treatments like monoclonal antibodies, and the wide availability of management guidelines, asthma control remained the major clinical challenge [33]. Added to the increased prevalence of asthma in adults (8.7%; S1 Fig in S1 Annex) in Ethiopia, the burden of uncontrolled asthma could be more throbbing to the already crippling health system.

This meta-analysis revealed based on the GINA asthma symptom control, 45.0%, 36%, and 19.0% of the asthma patients had uncontrolled, partially controlled, and controlled asthma in Ethiopia, respectively. Comparable findings were reported from a study done in European countries, where the rate of uncontrolled, partially controlled and controlled asthma was 45.0%, 34.8%, and 20.0% among adults, respectively [34]. In 2016, the Centers for Disease Control and Prevention (CDC) reported more than 60% of American adults with asthma had uncontrolled asthma [35]. Similarly, compared to the finding of this meta-analysis, a higher rate of uncontrolled asthma was reported in studies from France 48% [36], North Africa 50.9% [37], and Uganda 67.0% [38]. However, lower findings were reported from Brazil 34.2% [39], Morocco 29% [13], Japan 15.1% [40], Latin America (36.0%) [41], Saudi Arabia 38.0% [42], Sweden 39.6% [43], Middle East and North Africa 41.5% [44] and Asia-Pacific countries 30.0% [45]. These differences could be due to the geographical location, seasonal difference, number of the participants included, and the asthma service delivery at the institutions.

This meta-analysis also revealed a significant proportion of uncontrolled asthma in two major cities of Ethiopia, Jimma and Addis Ababa. It is obvious that these settings are responsible to manage a majority of the refractory cases. The increased proportion of uncontrolled asthma in these places was partly due to the presence of larger hospitals reserved for referral services in these regions. A body of evidence also showed geographical locations and urbanization paly a decisive role in determining the prevalence of asthma and its sensitivity to therapy [46, 47].

Based on the asthma control test (ACT), our review showed a higher rate of uncontrolled asthma in Ethiopia (78.0%). Similarly, a comparable finding was reported by Benkheder et al from North Africa [37] and Laforest et al from French [48], where 71.3% and 71.0% of the patients had uncontrolled asthma, respectively. However, in studies by Corrado et al from Italy [49], Zhong et al from China [50], Kabengele et al from Congo [51], and Stanford from the USA [52], relatively lower (51.3%, 55.1%, 56.0%, and 58%) of asthma patients had uncontrolled asthma, respectively. In Nigeria, a study reported 82.9% of the patients had uncontrolled asthma, even though the sample size of the study was small [53].

Several factors may contribute to poor disease control, with significant variability between different countries [54, 55]. Identifying these factors is fundamental for improving asthma outcomes. This systematic review revealed the most frequently reported predictors for uncontrolled asthma in Ethiopia were incorrect inhalation techniques, frequent SABA use, moderate/severe asthma, comorbid diseases, a history of asthma exacerbations and irregular follow-up.

Similarly, several studies have identified several factors associated with uncontrolled asthma. In studies by AL-Jahdali et al from Saudi Arabia [56], Bharti Chogtu et al from India [57], Fusun Yildiz et al from Turkey [58] and Umoh et al from Nigeria [53], improper use of asthma inhaler devices was associated with poorly controlled asthma. Studies from Sweden [59], Asia-Pacific countries [45] and Germany [60] revealed the overuse of short-acting beta-2 agonists (SABA) was associated with asthma exacerbations and asthma control. Price et al reported more than 40% of asthma patients used their reliever medications three or more times in the previous week, which the author may judge the reason for the high levels of uncontrolled asthma [34]. Several studies also reported patients with severe asthma [12, 61–63], history of asthma exacerbations/hospitalizations [45, 49, 53, 61, 63, 64], who had comorbid diseases [13, 50–52, 65], smoking [43, 52, 66], oral corticosteroid use [12, 63], and irregular follow-up visit [56, 67] were more likely had a higher rate of uncontrolled asthma.

Given the lack of previous attempts in producing such kind of aggregated data, the finding could hint at the adequacy of the health care service in dealing with the problem. It also provides valuable information for clinicians to design appropriate strategies that can help to provide proper care for asthmatic patients. The finding is also crucial for pharmacy professionals to deal with issues arising from improper use of asthma medication. Moreover, this finding could instigate researchers to further unlock matters related to such a huge burden of uncontrolled asthma in the current setting.

The major limitation of this review is the measurement of asthma control using GINA symptom control than lung function tests. This may overestimate the status of asthma control in the current settings. Heterogeneity, lack of uniformity for measuring asthma control, the inclusion of poor-quality study design, failure to identify specific groups with uncontrolled asthma, the inclusion of studies limited to a few geographic locations are some of the other shortcomings of this review.

## Conclusion

The rate of uncontrolled asthma in Ethiopia is much concerning. The most frequently reported predictors for uncontrolled asthma were; incorrect inhalation techniques, frequent SABA use, moderate/severe asthma, comorbid diseases, a history of asthma exacerbations and irregular follow-up visit. Revising the asthma management approaches and asthma educations at each follow-up visit should be strengthened to minimize the morbidity of uncontrolled asthma. Moreover, further research with a high-quality design is required to disclose the category of patients with a high proportion of uncontrolled asthma.

## Supporting information

S1 Annex (DOCX)

S1 Checklist PRISMA 2009 checklist. (DOC)

## References

[1] World Health Organization (WHO), “Asthma,” 2020. [Online]. Available: https://www.who.int/news-room/facts-in-pictures/detail/asthma.

[2] Buist A. S. and Vollmer W. M., “Reflections on the Rise in Asthma Morbidity and Mortality,” *JAMA J*. *Am*. *Med*. *Assoc*., vol. 264, no. 13, pp. 1719–1720, 1990, doi: 10.1001/jama.1990.03450130091034 2398613

[3] Anbesse Z. K., Mega T. A., Tesfaye B. T., and Negera G. Z., “Early readmission and its predictors among patients treated for acute exacerbations of chronic obstructive respiratory disease in Ethiopia: A prospective cohort study,” *PLoS One*, vol. 15, no. 10 October, pp. 16–29, 2020, doi: 10.1371/journal.pone.0239665 33022006 PMC7537865

[4] Stewart W. F., Ricci J. A., Chee E., and Morganstein D., “Lost Productive Work Time Costs from Health Conditions in the United States: Results from the American Productivity Audit,” *J*. *Occup*. *Environ*. *Med*., vol. 45, no. 12, pp. 1234–1246, 2003, doi: 10.1097/01.jom.0000099999.27348.78 14665809

[5] Sullivan P. W. et al., “The relationship between asthma, asthma control and economic outcomes in the United States,” *J*. *Asthma*, vol. 51, no. 7, pp. 769–778, 2014, doi: 10.3109/02770903.2014.906607 24697738

[6] Global Initiative for Asthma, “Global strategy for asthma management and prevention: Socioeconomics,” 2017. doi: 10.1016/S0335-7457(96)80056-6

[7] Demoly P. et al., “Prevalence of asthma control among adults in France, Germany, Italy, Spain and the UK,” *Eur*. *Respir*. *Rev*., vol. 18, no. 112, pp. 105–112, 2009, doi: 10.1183/09059180.00001209 20956130

[8] Demoly P., Annunziata K., Gubba E., and Adamek L., “Repeated cross-sectional survey of patient-reported asthma control in europe in the past 5 years,” *Eur*. *Respir*. *Rev*., vol. 21, no. 123, pp. 66–74, 2012, doi: 10.1183/09059180.00008111 22379176 PMC9487469

[9] Cazzoletti L. et al., “Asthma control in Europe: A real-world evaluation based on an international population-based study,” *J*. *Allergy Clin*. *Immunol*., vol. 120, no. 6, pp. 1360–1367, 2007, doi: 10.1016/j.jaci.2007.09.019 17981317

[10] Mintz M. et al., “Assessment of asthma control in primary care,” *Curr*. *Med*. *Res*. *Opin*., vol. 25, no. 10, pp. 2523–2531, 2009, doi: 10.1185/03007990903218655 19708765

[11] Peters S. P., Jones C. A., Haselkorn T., Mink D. R., Valacer D. J., and Weiss S. T., “Real-world Evaluation of Asthma Control and Treatment (REACT): Findings from a national Web-based survey,” *J*. *Allergy Clin*. *Immunol*., vol. 119, no. 6, pp. 1454–1461, 2007, doi: 10.1016/j.jaci.2007.03.022 17481716

[12] González Barcala F. J., de la Fuente-Cid R., Álvarez-Gil R., Tafalla M., Nuevo J., and Caamaño-Isorna F., “Factors associated with asthma control in primary care patients: The CHAS study,” *Arch*. *Bronconeumol*., vol. 46, no. 7, pp. 358–363, 2010, doi: 10.1016/j.arbres.2010.01.007 20227808

[13] Ghanname I. et al., “Factors associated with asthma control: MOSAR study (Multicenter Observational Study of Asthma in Rabat-Morocco),” *BMC Pulm*. *Med*., vol. 18, no. 1, pp. 1–13, 2018, doi: 10.1186/s12890-017-0557-5 29699541 PMC5921326

[14] Gebremariam T. H. et al., “Level of asthma control and risk factors for poor asthma control among clinic patients seen at a Referral Hospital in Addis Ababa, Ethiopia,” *BMC Res*. *Notes*, vol. 10, no. 1, pp. 4–9, 2017, doi: 10.1186/s13104-016-2343-5 29110731 PMC5674820

[15] Zewudie A., Nigussie T., Mamo Y., and Kumela K., “Determinants of poorly controlled asthma among asthmatic patients in Jimma University Medical Center, Southwest Ethiopia: A case control study,” *BMC Res*. *Notes*, vol. 12, no. 1, pp. 1–6, 2019, doi: 10.1186/s13104-018-4038-6 31429799 PMC6700762

[16] Moher D. et al., “Preferred reporting items for systematic reviews and meta-analyses: The PRISMA statement,” *PLoS Med*., vol. 6, no. 7, 2009, doi: 10.1371/journal.pmed.1000097 19621072 PMC2707599

[17] Abegaz T. M., Shegena E. A., Gessie N. F., Gebreyohannes E. A., and Seid M. A., “Barriers to and competency with the use of metered dose inhaler and its impact on disease control among adult asthmatic patients in Ethiopia.,” *BMC Pulm*. *Med*., vol. 20, no. 1, pp. 1–13, 2020. doi: 10.1186/s12890-019-1042-0 32085726 PMC7035747

[18] Downes M. J., Brennan M. L., Williams H. C., and Dean R. S., “Development of a critical appraisal tool to assess the quality of cross-sectional studies (AXIS),” *BMJ Open*, vol. 6, no. 12, pp. 1–7, 2016, doi: 10.1136/bmjopen-2016-011458 27932337 PMC5168618

[19] “Modified Newcastle—Ottawa Quality Assessment Scale,” [Online]. Available: https://bjsm.bmj.com/content/bjsports/51/23/1670/DC2/embed/inline-supplementary-material-2.pdf?download=true.

[20] Zemedkun K., Woldemichael K., and Tefera G., “Assessing control of asthma in Jush, Jimma, South West Ethiopia.,” *Ethiop*. *J*. *Health Sci*., vol. 24, no. 1, pp. 49–58, 2014, doi: 10.4314/ejhs.v24i1.7 24591799 PMC3929928

[21] Mebrahtom M., Mesfin N., Gebreyesus H., and Teweldemedhin M., “Status of metered dose inhaler technique among patients with asthma and its effect on asthma control in Northwest Ethiopia,” *BMC Res*. *Notes*, vol. 12, no. 1, pp. 1–6, 2019, doi: 10.1186/s13104-018-4038-6 30642378 PMC6332522

[22] Korinan Fanta F. B., “Asthmatic Patients on Follow-Up At Chest Clinic of Jimma University,” *Indo Am*. *J*. *Pharm*. *Res*., vol. 6, no. 11, pp. 2231–6876, 2016.

[23] Tesfaye Tsegaye T. B., Bruck Messele, Gebremedhin Beedemariamand A, “Asthma Treatment Outcome and Factors Associated with Uncontrolled Asthma among Adult Asthmatic Patients Attending Ambulatory Care Units of Selected Public Hospitals in Addis Ababa, Ethiopia,” pp. 15–16, 2019.

[24] Dalo A., Yarlagadda R., and Tatiparthi R., “Assessment of Asthma Treatment Outcomes among Adult Outpatients at Nemmh Chest Clinic in Hadiya Zone, Southern,” *Pulsus*, pp. 132–137, 2017.

[25] Zeru T. G., Engidawork E., and Berha A. B., “Assessment of Asthma Control and Quality of Life among Asthmatic Patients Attending Armed Forces Referral and Teaching Hospital, Addis Ababa, Ethiopia,” *Pulm*. *Med*., vol. 2020, 2020, doi: 10.1155/2020/5389780 32802503 PMC7411494

[26] Woledesenbet M. A., Shumet Mekonen S., Sori L. M., and Abegaz T. M., “Epidemiology of Depression and Associated Factors among Asthma Patients in Addis Ababa, Ethiopia,” *Psychiatry J*., vol. 2018, pp. 1–7, 2018, doi: 10.1155/2018/5934872 30225243 PMC6129350

[27] Kebede B., Mamo G., and Molla A., “Association of Asthma Control and Metered-Dose Inhaler Use Technique among Adult Asthmatic Patients Attending Outpatient Clinic, in Resource-Limited Country: A Prospective Study,” *Can*. *Respir*. *J*., vol. 2019, 2019, doi: 10.1155/2019/6934040 31467621 PMC6701327

[28] Pawankar R., Canonica G. W., Holgate S. T., and Lockey R. F., “Allergic diseases and asthma: A major global health concern,” *Curr*. *Opin*. *Allergy Clin*. *Immunol*., vol. 12, no. 1, pp. 39–41, 2012, doi: 10.1097/ACI.0b013e32834ec13b 22157151

[29] Beran D., Zar H. J., Perrin C., Menezes A. M., and Burney P., “Burden of asthma and chronic obstructive pulmonary disease and access to essential medicines in low-income and middle-income countries,” *Lancet Respir*. *Med*., vol. 3, no. 2, pp. 159–170, 2015, doi: 10.1016/S2213-2600(15)00004-1 25680912

[30] Nunes C., Pereira A. M., and Morais-Almeida M., “Asthma costs and social impact,” *Asthma Res*. *Pract*., vol. 3, no. 1, pp. 1–11, 2017, doi: 10.1186/s40733-016-0029-3 28078100 PMC5219738

[31] Adeloye D., Chan K. Y., Rudan I., and Campbell H., “An estimate of asthma prevalence in Africa: A systematic analysis,” *Croat*. *Med*. *J*., vol. 54, no. 6, pp. 519–531, 2013, doi: 10.3325/cmj.2013.54.519 24382846 PMC3893990

[32] Bousquet J., Bousquet P. J., Godard P., and Daures J. P., “The public health implications of asthma,” *Bull*. *World Health Organ*., vol. 83, no. 7, pp. 548–554, 2005, doi: 10.1590/S0042-96862005000700016 16175830 PMC2626301

[33] Gupta R. S., Carrión-Carire V., and Weiss K. B., “The widening black/white gap in asthma hospitalizations and mortality,” *J*. *Allergy Clin*. *Immunol*., vol. 117, no. 2, pp. 351–358, 2006, doi: 10.1016/j.jaci.2005.11.047 16461136

[34] Price D., Fletcher M., and Van Der Molen T., “Asthma control and management in 8,000 European patients: The REcognise Asthma and LInk to Symptoms and Experience (REALISE) survey,” *NPJ Prim*. *Care Respir*. *Med*., vol. 24, no. October 2013, pp. 1–10, 2014, doi: 10.1038/npjpcrm.2014.9 24921985 PMC4373302

[35] Centers for Disease Control and Prevention (CDC), “Uncontrolled Asthma among Adults, 2016,” p. 2016, 2016.

[36] Raherison C. et al., “Asthmatic patient: control, feeling and compliance. French results,” 2016.

[37] Benkheder A. et al., “Control of asthma in the Maghreb: results of the AIRMAG study,” *Respir*. *Med*., vol. 103, no. SUPPL. 2, pp. S12–S20, 2009, doi: 10.1016/S0954-6111(09)70023-X 20122624

[38] Serugendo A. N., Kirenga B. J., Hawkes M., Nakiyingi L., Worodria W., and Okot-Nwang M., “Evaluation of asthma control using Global Initiative for Asthma criteria and the Asthma Control Test in Uganda,” *Int*. *J*. *Tuberc*. *Lung Dis*., vol. 18, no. 3, pp. 371–376, 2014, doi: 10.5588/ijtld.13.0699 24670578

[39] Gazzotti M. R., Nascimento O. A., Montealegre F., Fish J., and Jardim J. R., “Level of asthma control and its impact on activities of daily living in asthma patients in Brazil,” vol. 39, no. February, pp. 532–538, 2013. doi: 10.1590/S1806-37132013000500002 24310625 PMC4075876

[40] Adachi M., Hozawa S., Nishikawa M., Yoshida A., Jinnai T., and Tamura G., “Asthma control and quality of life in a real-life setting: a cross-sectional study of adult asthma patients in Japan (ACQUIRE-2),” *J*. *Asthma*, vol. 56, no. 9, pp. 1016–1025, 2019, doi: 10.1080/02770903.2018.1514628 30252543

[41] Gold L. S. et al., “Level of asthma control and healthcare utilization in Latin America,” *Allergy Eur*. *J*. *Allergy Clin*. *Immunol*., vol. 68, no. 11, pp. 1463–1466, 2013, doi: 10.1111/all.12237 24117970

[42] Hamdan AL-Jahdali and T. D., Siraj Wali, Gamal Salem, Fahad Al-Hameed, Abdullah Almotair, Mohammed Zeitouni et al, “Asthma control and predictive factors among adults in Saudi Arabia: Results from the Epidemiological Study on the Management of Asthma in Asthmatic Middle East Adult Population study,” *Ann*. *Thorac*. *Meedicine*, vol. 14, no. 2, pp. 148–154, 2019, doi: 10.4103/atm.ATM PMC6467022 31007767

[43] Ställberg B., Lisspers K., Hasselgren M., Janson C., Johansson G., and Svärdsudd K., “Asthma control in primary care in Sweden: A comparison between 2001 and 2005,” *Prim*. *Care Respir*. *J*., vol. 18, no. 4, pp. 279–286, 2009, doi: 10.4104/pcrj.2009.00024 19455269 PMC6619358

[44] Tarraf H. et al., “Asthma control in adults in the Middle East and North Africa: Results from the ESMAA study,” *Respir*. *Med*., vol. 138, no. November 2017, pp. 64–73, 2018, doi: 10.1016/j.rmed.2018.03.024 29724395

[45] Gold L. S., Thompson P., Salvi S., Faruqi R. A., and Sullivan S. D., “Level of asthma control and health care utilization in Asia-Pacific countries,” *Respir*. *Med*., vol. 108, no. 2, pp. 271–277, 2014, doi: 10.1016/j.rmed.2013.12.004 24406243

[46] Rodriguez A., Brickley E., Rodrigues L., Normansell R. A., Barreto M., and Cooper P. J., “Urbanisation and asthma in low-income and middle-income countries: a systematic review of the urban-rural differences in asthma prevalence,” *Thorax*, vol. 74, no. 11, pp. 1020–1030, 2019, doi: 10.1136/thoraxjnl-2018-211793 31278168 PMC6860411

[47] Jauhiainen A. et al., “Impact of season and geography on CompEx Asthma: a composite end-point for exacerbations,” *ERJ Open Res*, no. July, 2020, doi: 10.1183/23120541.00246–2020 PMC7569167 33123561

[48] Laforest L. et al., “Asthmatic patients’ poor awareness of inadequate disease control: A pharmacy-based survey,” *Ann*. *Allergy*, *Asthma Immunol*., vol. 98, no. 2, pp. 146–152, 2007, doi: 10.1016/S1081-1206(10)60687-4 17304881

[49] Corrado A., Renda T., Polese G., and Rossi A., “Assessment of asthma control: The SERENA study,” *Respir*. *Med*., vol. 107, no. 11, pp. 1659–1666, 2013, doi: 10.1016/j.rmed.2013.08.019 24045118

[50] Zhong N. et al., “Uncontrolled asthma and its risk factors in adult Chinese asthma patients,” *Ther*. *Adv*. *Respir*. *Dis*., vol. 10, no. 6, pp. 507–517, 2016, doi: 10.1177/1753465816663978 27595644 PMC5933594

[51] Kabengele B. O., Kayembe J. M. N., Kayembe P. K., Kashongue Z. M., Kaba D. K., and Akilimali P. Z., “Factors associated with uncontrolled asthma in adult asthmatics in Kinshasa, Democratic Republic of Congo,” *PLoS One*, vol. 14, no. 4, pp. 1–13, 2019, doi: 10.1371/journal.pone.0215530 30998727 PMC6472784

[52] Stanford R. H., Gilsenan A. W., Ziemiecki R., Zhou X., Lincourt W. R., and Ortega H., “Predictors of uncontrolled asthma in adult and pediatric patients: Analysis of the asthma control characteristics and prevalence survey studies (ACCESS),” *J*. *Asthma*, vol. 47, no. 3, pp. 257–262, 2010, doi: 10.3109/02770900903584019 20210612

[53] Umoh V., Ekott J., Ekpo O., and Ekwere M., “Asthma control among patients in Uyo South-Eastern Nigeria,” *Indian J*. *Allergy*, *Asthma Immunol*., vol. 27, no. 1, p. 27, 2013, doi: 10.4103/0972-6691.116611

[54] Tamara Lourido-Cebreiro C. R.-G., David Facal and F.-J. Gonzalez-Barcala, “Asthma Control: A continuing Challenge,” vol. 1, no. 2, pp. 7–13, 2019.

[55] Braido F., “Failure in Asthma Control: Reasons and Consequences,” *Scientifica (Cairo)*., vol. 2013, pp. 1–15, 2013, doi: 10.1155/2013/549252 24455432 PMC3881662

[56] AL-Jahdali H. et al., “Improper inhaler technique is associated with poor asthma control and frequent emergency department visits,” *Allergy*, *Asthma Clin*. *Immunol*., vol. 9, no. 1, pp. 1–7, 2013, doi: 10.1186/1710-1492-9-8 23510684 PMC3605255

[57] Chogtu B., Holla S., Magazine R., and Kamath A., “Evaluation of relationship of inhaler technique with asthma control and quality of life,” *Indian J*. *Pharmacol*., vol. 49, no. 1, pp. 110–115, 2017, doi: 10.4103/0253-7613.201012 28458433 PMC5351222

[58] Yildiz F. et al., “Importance of inhaler device use status in the control of asthma in adults: The asthma inhaler treatment study,” *Respir*. *Care*, vol. 59, no. 2, pp. 223–230, 2014, doi: 10.4187/respcare.02478 23882109

[59] Nwaru B. I., Ekström M., Hasvold P., Wiklund F., Telg G., and Janson C., “Overuse of short-acting β2-agonists in asthma is associated with increased risk of exacerbation and mortality: A nationwide cohort study of the global SABINA programme,” *Eur*. *Respir*. *J*., vol. 55, no. 4, 2020, doi: 10.1183/13993003.01872-2019 31949111 PMC7160635

[60] Worth H. et al., “Prevalence of overuse of short-acting beta-2 agonists (SABA) and associated factors among patients with asthma in Germany,” *Respir*. *Res*., vol. 22, no. 1, pp. 1–8, 2021, doi: 10.1186/s12931-020-01578-8 33863317 PMC8051057

[61] Neffen H. et al., “Key factors associated with uncontrolled asthma–the Asthma Control in Latin America Study,” *J*. *Asthma*, vol. 57, no. 2, pp. 113–122, 2020, doi: 10.1080/02770903.2018.1553050 30915868

[62] Albataineh E., Al-Zayadneh E., Al-Shagahin H., AL Soloman A., Altarawneh A., and Aldmour I., “Asthma Control and Its Predictive Factors in Adult Asthma Patients,” *J*. *Clin*. *Med*. *Res*., vol. 11, no. 12, pp. 807–817, 2019, doi: 10.14740/jocmr4021 31803325 PMC6879035

[63] Turktas H., Mungan D., Uysal M. A., and Oguzulgen K., “Determinants of asthma control in tertiary level in Turkey: A cross-sectional multicenter survey,” *J*. *Asthma*, vol. 47, no. 5, pp. 557–562, 2010, doi: 10.3109/02770901003692777 20560829

[64] Enríquez-Matas A., Fernández-Rodríguez C., Andrés Esteban E. M., and Fernández-Crespo J., “Main contributory factors on asthma control and health-related quality of life in elderly asthmatics,” *J*. *Investig*. *Allergol*. *Clin*. *Immunol*., vol. 30, no. 4, pp. 264–271, 2020, doi: 10.18176/jiaci.0430 31283523

[65] Sastre J. et al., “Anxiety, Depression, and Asthma Control: Changes After Standardized Treatment,” *J*. *Allergy Clin*. *Immunol*. *Pract*., vol. 6, no. 6, pp. 1953–1959, 2018, doi: 10.1016/j.jaip.2018.02.002 29454162

[66] Abrahamsen R., Gundersen G. F., Svendsen M. V., Klepaker G., Kongerud J., and Fell A. K. M., “Possible risk factors for poor asthma control assessed in a cross-sectional population-based study from Telemark, Norway,” *PLoS One*, vol. 15, no. 5, pp. 1–14, 2020, doi: 10.1371/journal.pone.0232621 32396562 PMC7217450

[67] Park H. J. et al., “Regular follow-up visits reduce the risk for asthma exacerbation requiring admission in Korean adults with asthma,” *Allergy*, *Asthma Clin*. *Immunol*., vol. 14, no. 1, pp. 1–7, 2018, doi: 10.1186/s13223-018-0250-0 30002684 PMC6038276

