# Supplementary material for: Asthma control and its predictors in Ethiopia: Systematic review and meta-analysis
Source: PLoS One. 2022 Jan 13;17(1):e0262566. doi: 10.1371/journal.pone.0262566 (PMC8758033; doi:10.1371/journal.pone.0262566)
Supplement: S1 Annex — (DOCX) [file pone.0262566.s001.docx]

**ANNEX A**

S1 Table: Appraisal of Cross-sectional Studies (AXIS)

| **Question** | | Gebremariam | Zemdekun | Tsegaye | Woldesenbet | Fanta | Dalo | Abegaz | Mebrahtom | Zeru |
| --- | --- | --- | --- | --- | --- | --- | --- | --- | --- | --- |
| **Introduction** | | | |  | | | | | | |
| 1 | Were the aims/objectives of the study clear? | Yes | Yes | Yes | Yes | Yes | Yes | Yes | Yes | Yes |
| **Methods** | | | |  |  |  |  |  |  |  |
| 2 | Was the study design appropriate for the stated aim(s)? | Yes | Yes | Yes | Yes | Yes | Yes | Yes | Yes | Yes |
| 3 | [Was the sample size justified?](#_bookmark6) | Yes | Yes | Yes | Yes | No | Yes | Yes | Yes | Yes |
| 4 | Was the target/reference population clearly defined? (Is it clear who the [research was about?)](#_bookmark8) | Yes | Yes | Yes | Yes | Yes | Yes | Yes | Yes | Yes |
| 5 | Was the sample frame taken from an appropriate population base so that it [closely represented the target/reference population under investigation?](#_bookmark9) | Yes | Yes | Yes | Yes | Yes | Yes | Yes | Yes | Yes |
| 6 | [Was the selection process likely to select subjects/participants that were](#_bookmark11) representative of the target/reference population under investigation? | Yes | Yes | Yes | No | No | Yes | No | Yes | No |
| 7 | Were measures undertaken to address and categorise non-responders? | No | No | Yes | No | No | NA | No | No | No |
| 8 | Were the risk factor and outcome variables measured appropriate to the aims [of the study?](#_bookmark13) | Yes | Yes | Yes | Yes | Yes | Yes | Yes | Yes | Yes |
| 9 | Were the risk factor and outcome variables measured correctly using instruments/measurements that had been trialled, piloted or published [previously?](#_bookmark13) | Yes | Yes | Yes | Yes | Yes | No | Yes | Yes | Yes |
| 10 | Is it clear what was used to determined statistical significance and/or precision estimates? (e.g. p-values, confidence intervals) | Yes | Yes | Yes | Yes | Yes | Yes | Yes | Yes | Yes |
| 11 | Were the methods (including statistical methods) sufficiently described to [enable them to be repeated?](#_bookmark16) | Yes | Yes | Yes | Yes | Yes | No | Yes | Yes | Yes |
| **Results** | | | |  |  |  |  |  |  |  |
| 12 | Were the basic data adequately described? | yes | Yes | Yes | Yes | Yes | Yes | Yes | Yes | Yes |
| 13 | Does the response rate raise concerns about non-response bias? | Yes | No | Yes | Yes | No | NA | No | Yes | Yes |
| 14 | If appropriate, was information about non-responders described? | Yes | No | Yes | No | No | NA | No | No | Yes |
| 15 | Were the results internally consistent? | Yes | Yes | Yes | Yes | Yes | Yes | Yes | Yes | Yes |
| 16 | Were the results presented for all the analyses described in the methods? | Yes | yes | Yes | Yes | Yes | Yes | Yes | Yes | Yes |
| **Discussion** | | | |  |  |  |  |  |  |  |
| 17 | Were the authors’ discussions and conclusions justified by the results? | Yes | Yes | Yes | Yes | Yes | Yes | Yes | Yes | Yes |
| 18 | Were the limitations of the study discussed? | Yes | Yes | Yes | Yes | No | Yes | Yes | Yes | Yes |
| **Others** | | | |  |  |  |  |  |  |  |
| 19 | Were there any funding sources or conflicts of interest that may affect the authors’ interpretation of the results? | Yes | Yes | No | Yes | yes | No | Yes | Yes | Yes |
| 20 | Was ethical approval or consent of participants attained? | Yes | Yes | Yes | Yes | Yes | Yes | Yes | Yes | Yes |

NA: not applicable

S2 Table: Modified Newcastle-Ottawa Quality Assessment Scale for the included observational studies

| Selection^a^ | | | | | Comparability^b^ | Outcome^c^ | | | |
| --- | --- | --- | --- | --- | --- | --- | --- | --- | --- |
| Included Studies | Representativeness of  the exposed cohort | Selection of non- Exposed | Ascertainment of exposure | Outcome of interest was not present at start of study |  | Assessment of Outcome | Length of Follow-up | Adequacy of Follow-up | Total number of stars^d^ |
| Kebede | A * | A* | B* | A* | C | A* | A* | A* | 7 |
| Zewdie | A* | A* | B* | B | C | A* | A* | A* | 6 |
| **^a^Selection**:  (1) Representativeness of the exposed cohort: A) Consecutive eligible participants were selected, participants were randomly selected, or all participants were invited to participate from the source population, B) Not satisfying requirements in part (a), or not stated.  (2) Selection of the non-exposed cohort: A, Selected from the same source population* B) Selected from a different source population C) No description. (3) Ascertainment of exposure: A) Structured injury data (e.g. record completed by medical staff)* B) Structured interview* C) Written self-report D) No description  (4) For a demonstration that the outcome of interest was not present at the start of the study: A) Yes* B) No or not explicitly stated  **Comparability**: For comparability of cohorts based on the design or analysis:  A) Study controls for previous injury*  B) Study controls for age*, sex  C) Not comparable for confounders  **^c^Outcome**:  (1) Assessment of outcome: A) Independent or blind assessment stated, or confirmation of the outcome by reference to secure records (e.g. imaging, structured injury data, etc.)* B) record linkage (e.g. identified through ICD codes on database records)* C) Self-report with no reference to original structured injury data or imaging D) No description  2) Was follow-up long enough for outcomes to occur?  A) Yes (≥3 months)* B) No (<3 months)  3) Adequacy of follow up of cohorts: A) Complete follow up – all participants accounted for* B) Subjects lost to follow up unlikely to introduce bias (<15% lost to follow up, or description provided of those lost*) C) Follow up rate <85% and no description of those lost provided D) No statement  ^d^ Total is out of 9 stars  Note: > 7 High-quality study; 5-7 Moderate quality study; <5 Low-quality study | | | | | | | | | |

S3 Table: PubMed Search details

| #1 | Asthma[MeSH Terms] OR Asthma[tw] OR "bronchial asthma"[tw] |
| --- | --- |
| #2 | "Prevention and control"[MeSH Subheading] OR control[tw] OR controlled[tw] OR uncontrolled[tw] |
| #3 | Ethiopia[MeSH Terms] OR Ethiopia[tw] |
| #4 | #1 AND #2 AND #3 |

**Annex B: Figures for the prevalence of asthma and COPD among adults in Ethiopia**


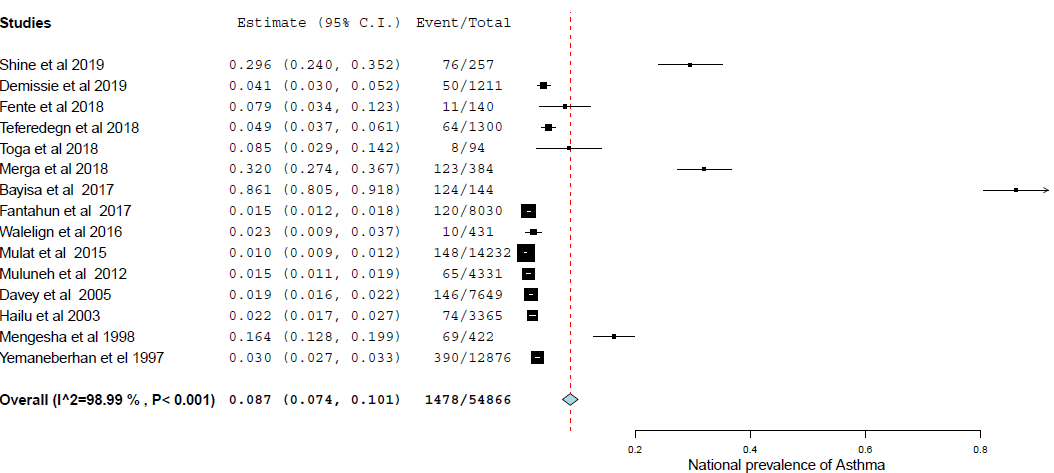


S1 Fig.: Overall prevalence of Asthma in Ethiopia


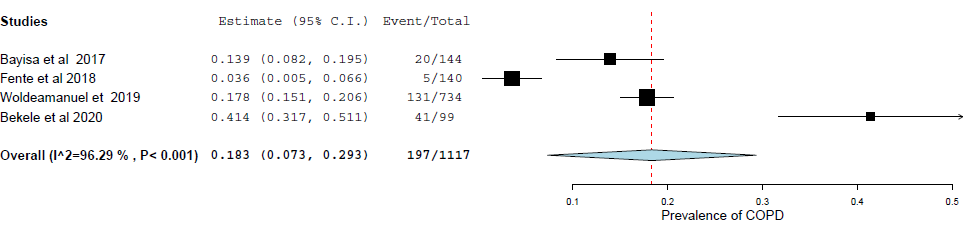


S2 Fig.: Overall prevalence of COPD in Ethiopia
